# Quantitative Evaluation of the Blending Between Virgin and Aged Aggregates in Hot-Mix Recycled Asphalt Mixtures

**DOI:** 10.3390/ma18235439

**Published:** 2025-12-02

**Authors:** Haoyang Zou, Yunlong Sui, Wei Lu, Teng Wang, Dedong Guo, Xupeng Sun, Zhiye Liu

**Affiliations:** 1 Shandong Key Laboratory of Technologies and Systems for Intelligent Construction Equipment, Shandong Jiaotong University, Ji’nan 250357, China; 24107058@stu.sdjtu.edu.cn (H.Z.); tw@chd.edu.cn (T.W.); guodedong@sdjtu.edu.cn (D.G.); 22107029@stu.sdjtu.edu.cn (X.S.); 24107025@stu.sdjtu.edu.cn (Z.L.); 2 Postdoctoral Workstation of Beijing Road Engineering Quality Supervision Station, Beijing 100071, China; 3 Jinan Urban Construction Group, Ji’nan 250031, China

**Keywords:** plant hot-mix recycled asphalt mixes, recycled aggregate particles, degree of blending, mixing condition, magnetite aggregate

## Abstract

Severe asphalt ageing and the difficulty in dispersing agglomerated particles within reclaimed asphalt pavement (RAP) hinder the uniform blending of virgin and aged mineral aggregates during plant-mixed hot recycling, compromising the durability of the recycled asphalt mixture. To accurately quantify the degree of blending between virgin and aged aggregate during thermal recycling and to optimise the mix design and mixing process for thermally recycled asphalt mixtures, a test method has been proposed. This method comprises key steps, including the preparation of asphalt mixtures, separation of virgin and aged materials, separation of the binder from aggregate, and calculation of the blending degree. It analyses the impact of varying mixing conditions on the blending degree of virgin and aged aggregate during the thermal recycling process. The results indicate that complete homogenization of virgin and aged aggregates during mixing is unattainable, with blending efficiency ranging from 40% to 60%. Increasing the amount of RAP has a negligible effect on blending efficiency. Appropriate increases in the amount of rejuvenating agent, mixing temperature, mixing time, and asphalt content enhance blending efficiency by 10% to 30%. The mixing sequence where RAP is first blended with virgin aggregate before incorporating virgin asphalt further enhances the blending efficiency of virgin and aged aggregates by approximately 20%. However, mixing temperatures exceeding 160 °C and mixing times exceeding 270 s caused secondary ageing of the asphalt, adversely affecting the blending degree of virgin and aged aggregates.

## 1. Introduction

Most high-grade highways worldwide adopt asphalt pavement structures. Due to the combined effects of external environmental factors and traffic loads during service, functional and structural defects gradually develop. During the reconstruction and maintenance of asphalt pavements, large quantities of reclaimed asphalt pavement (RAP) are generated [1,2,3]. On the one hand, RAP directly occupies limited land, reducing land use efficiency and impacting the surrounding ecological environment. On the other hand, the loss of high-quality aggregate resources such as basalt, diabase, and andesite increases the demand pressure for new materials. It leads to economic losses in road construction and maintenance [4,5]. RAP containing aged aggregate and asphalt can be reused in the mixing of asphalt mixtures, achieving its recycling through asphalt pavement recycling technology [6]. The global recycling rate of RAP has reached 75% to 100%, playing a significant role in energy conservation, carbon reduction, and ecological and environmental protection. Among these methods, plant-mixed hot recycling technology demonstrates the highest quality utilisation of RAP and is the most widely adopted [7,8].

**Figure 1:**
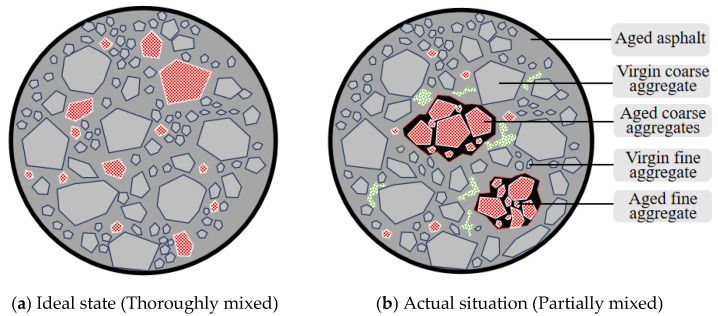
Two different mixed-state diagrams.

Plant-mixed hot-recycled asphalt mixture typically consists of 20% to 30% RAP by mass, combined with virgin asphalt and virgin aggregate. The aggregate gradation design for hot-recycled mixes is fundamentally consistent with conventional asphalt mixes. The gradation of RAP is determined through extraction and sieve analysis tests, assuming that during mixing, the aged aggregate in RAP can be freed from asphalt binder and uniformly blended with virgin aggregate, representing an ideal state (Figure 1a). However, since the coarse and fine aggregate particles in RAP are tightly bonded by aged asphalt and form agglomerates, the mixing time for the hot-recycled mix is only about 1 min. This prevents the fine-grained recycled aggregate particles coated with asphalt from fully separating from the coarse-grained recycled aggregate particles during mixing [9]. They also cannot be uniformly blended with the virgin aggregate. Consequently, the actual aggregate gradation of the hot-recycled asphalt mix deviates from the design gradation—reflecting the actual situation(Figure 1b). This outcome will result in substandard compaction of the thermally RAP, leading to an excessively high permeability coefficient. This predisposes the surface to premature deterioration, adversely affecting the road’s service life [10,11].

Domestic and international scholars have conducted in-depth and sustained research into the uniformity of aggregate distribution in standard asphalt mixtures. Chen et al. [12] investigated the influence of aggregate gradation uniformity on pavement service life and performance characteristics through an experimental method involving the sieving of asphalt mixtures after combustion. Zhang et al. [13] employed MATLAB image processing techniques to evaluate the uniformity of aggregate distribution in asphalt pavements at both surface and internal structural levels. This was achieved by capturing surface texture images and cross-sectional images of pavement cores. Liu et al. [14] employed steel slag with a 5 mm to 10 mm particle size to replace the original aggregate. Utilising a U-Net network to localise the steel slag, they achieved rapid and accurate analysis of aggregate dispersion uniformity within the asphalt mixture, thereby supporting construction inspection in road engineering. Domestic research on asphalt mixture uniformity predominantly focuses on digital image processing and computed tomography (CT) techniques. Peng Yong et al. [15,16,17] applied digital image processing to mixture uniformity studies by comprehensively considering aggregates’ spatial and quantitative distribution within the mixture. Li Shenglian et al. [18] investigated the factors influencing the paving uniformity of asphalt mixtures by comparing different aggregate gradations, light intensity levels, and image acquisition heights using digital image processing techniques. Guo Naisheng et al. [19] conducted experiments with three gradations and four asphalt designs, proposing uniformity evaluation metrics for single-layer cross-sections and individual specimens of asphalt mixtures. They assessed mixture uniformity by identifying internal specimen structures through CT technology.

Numerous scholars have also investigated the mixing state of virgin and aged aggregates in asphalt recycled mixtures using methods such as Fourier transform infrared spectroscopy (FTIR), fluorescent powder addition, and equivalent diameter [20,21,22]. However, the impact of these mixtures on the gradation uniformity and compaction quality of asphalt hot-recycled mixtures has not received sufficient attention. Navaro et al. [23] prepared reclaimed asphalt concrete micro-samples via compaction, cutting, and polishing to observe RAP aggregate distribution at the microscopic level, analysing the asphalt mixture recycling process. At the macroscopic level, Chen et al. [12] employed the cross-uniform distribution method to verify the lateral uniformity of asphalt and aggregates, using a four-layer sampling technique to evaluate longitudinal uniformity. With the increasing application of digital image processing in observation, Gong et al. [24] combined this technique with representative volume elements to study the contact characteristics and mortar distribution within thermally regenerated asphalt mixtures. This technique indirectly characterised the homogeneity of the asphalt mixture and analysed its key influencing factors. Tang et al. [25] proposed a novel regionalisation method based on digital image processing to more directly and accurately evaluate the influence of the amount of RAP, agglomeration, and asphalt type on aggregate dispersion uniformity. Tang et al. [26] employed white dolomite as fresh aggregate, which was mixed with RAP, formed, cut, and subsequently analysed using digital image processing (DIP) technology. The effects of refined disintegration and conventional crushing pretreatment methods, along with the incorporation rate of RAP, on the uniformity of virgin aggregates were analysed. Yu Zhilong et al. [27] simulated asphalt recycled mixtures with varying degrees of segregation through laboratory testing, introducing uniformity as a metric for evaluating road performance. Ma Tao et al. [28] investigated the dispersion of aged asphalt mixtures through laboratory mixing and sieving tests, proposing improvement strategies concerning heating temperature, mixing duration, and rejuvenator dosage.

Existing research has confirmed that virgin and aged aggregates exhibit varying degrees of blending behaviour during the thermal recycling process, though most studies remain largely descriptive in nature. It is widely recognised that the pavement performance of asphalt mixtures is influenced by multiple objective factors, rather than being solely determined by the degree of blending between virgin and aged aggregates. Furthermore, existing research methodologies, such as DIP techniques, binder tracing methods, and FTIR, commonly suffer from operational complexity, imprecise blending models, a disconnect from actual production processes, and difficulties in quantitative analysis. To quantitatively analyse the mixing state of aged aggregate particles, this paper proposes a test method for determining and analysing the movement changes in aged aggregate particles within RAP during the hot recycling process, based on the practical application of plant-mixed hot recycling. Through experimentation, it examines the mixing state and patterns of virgin and aged aggregate particles across different particle sizes under six influencing factors: amount of RAP, amount of rejuvenating agent, mixing temperature, mixing time, mixing order, and asphalt content.

## 2. Materials and Experimental Design

### 2.1. Materials

#### 2.1.1. Virgin Asphalt

This study employed 70# matrix asphalt as the virgin asphalt. The technique performances of virgin asphalt were tested according to the Chinese specification of Standard Test Methods of Bitumen and Bituminous Mixtures for Highway Engineering (JTG E20–2011) [29], with the results presented in Table 1.

#### 2.1.2. Virgin Aggregates

**Figure 2:**
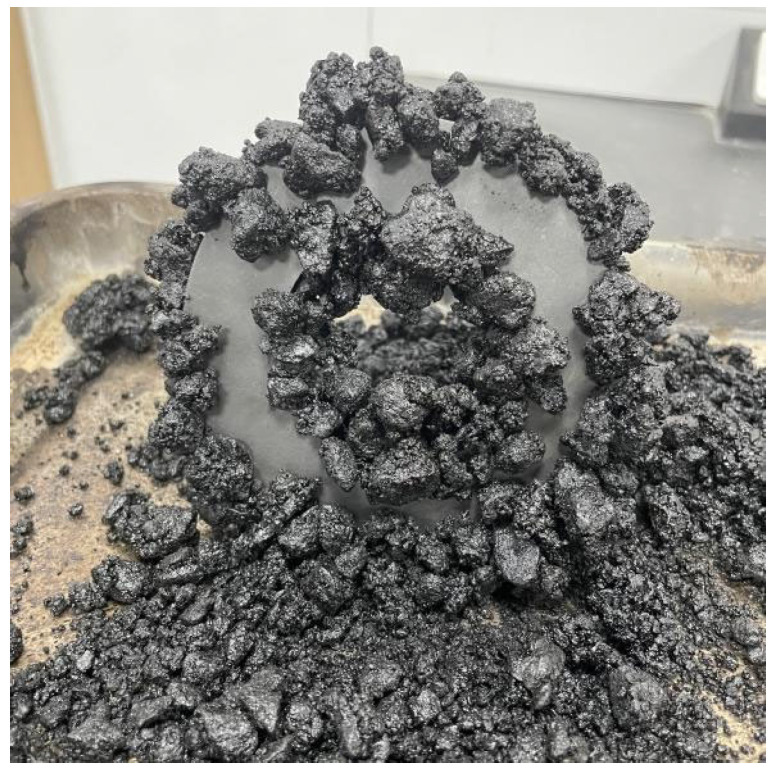
Magnetite aggregate.

Coarse aggregate was selected from the magnetite ore matrix sourced from Tongling, Anhui Province. After crushing and screening, magnetite aggregates of 5 to 10 mm and 10 to 20 mm sizes were obtained. As illustrated in Figure 2, the mixture prepared with it can be attracted by a magnet.

As shown in Table 2, testing has confirmed that all technical specifications of magnetite aggregate meet the quality requirements for coarse aggregate in asphalt pavements stipulated by ministerial standards. Compared to ordinary aggregates, magnetite aggregate exhibits a higher density, superior strength, greater hardness, and enhanced wear resistance [30]. Its crystal structure gives rise to numerous spontaneously magnetised domains within its structure. When subjected to an external magnetic field, these magnetic domains align in an orderly fashion, generating a powerful attraction that enables them to be drawn towards permanent magnets. To eliminate the effects of dust and other contaminants adhering to the aggregate surface, the material is first washed with water and then dried.

In this study, the 4.75 to 9.5 mm aggregate fraction was substituted with magnetite aggregate, while all other sizes of mineral aggregate comprised limestone.

#### 2.1.3. RAP

The RAP was screened and classified into two specifications based on particle size: 0 to 10 mm and 10 to 20 mm. Following extraction and screening, the asphalt content measured in the aforementioned RAP was 4.5% and 3.0%, respectively. Whilst the aged asphalt was obtained by extraction from RAP sourced from a specific location in Zhangqiu, Jinan, Shandong Province. The results of the aged asphalt performance testing are shown in Table 3, and the single-graded aggregates used in the test are shown in Table 4.

### 2.2. Experimental Procedure

To accurately analyse the mixing state of virgin and aged aggregate during the mixing process of hot-recycled asphalt pavement mixes, a method for determining aggregate mixing uniformity is proposed herein. This method assesses the dispersion uniformity of aged aggregate during mixing by comparing the movement characteristics of fine-grained aggregate in RAP under actual versus ideal conditions.

**Figure 3:**
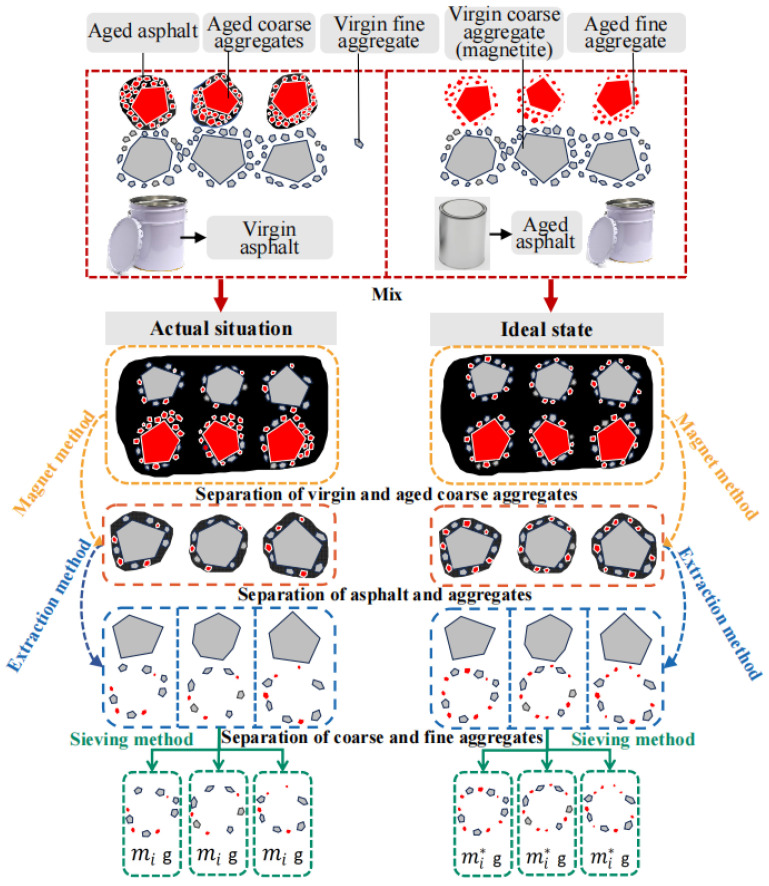
Schematic design.

The fundamental principle of this test is illustrated in Figure 3. For comparative analysis, two types of hot-recycled mixtures must be prepared: one under actual conditions and one under ideal conditions. The actual hot-recycled mixture is produced by mixing RAP, virgin asphalt, and virgin aggregate, with magnetite aggregate serving as the coarse aggregate. Since RAP exists in an agglomerated state, it must be fully dispersed and thoroughly mixed with virgin aggregate during mixing. To address this issue, the ideal thermal recycled mixture is produced by blending new asphalt, new aggregate, and RAP that has undergone oil–aggregate separation through extraction and recovery testing, together with used asphalt and used aggregate, with magnetite aggregate again serving as the coarse aggregate. Both mixes exhibit identical gradation and binder-to-aggregate ratios. Magnetically adhered fine-grained mineral particles are separated from the magnetite aggregate via magnetic attraction. Following extraction and screening, the particle size distribution and quantity of surface mineral particles on the magnetite aggregate are compared against the ideal state. This analysis reveals the true mixing state of the aged mineral particles within the RAP during the thermal recycling process.

**Figure 4:**
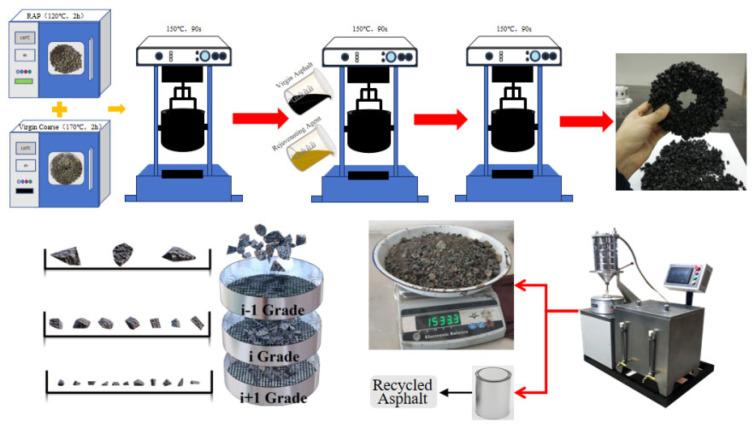
Process flow chart for preparation.

The specific test process can be divided into four parts: preparing asphalt mixtures, separating virgin and aged materials, separating asphalt from aggregates, and calculating the blending degree. The mixture preparation procedure is shown in Figure 4.

#### 2.2.1. Preparing Asphalt Mixtures

(1) Actual situation:

① Calculate and weigh the mass of various aggregates, binders, and other raw materials according to the mix design. Place the RAP in a 120 °C constant-temperature drying oven for heating treatment. Place the mineral powder and virgin aggregates in a 170 °C constant-temperature drying oven for heating treatment. Heat the virgin asphalt in a 150 °C oven until it reaches a fluid state.

**Figure 5:**
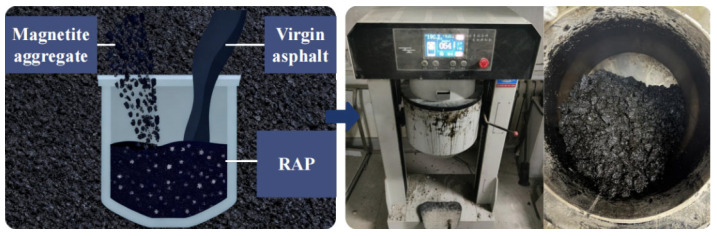
Mixing Process Diagram.

② Place virgin aggregate and RAP into a 150 °C mixing drum and mix for 90 s. Then add asphalt and rejuvenator and mix for 90 s. Finally, add mineral powder and mix for another 90 s, as shown in Figure 5.

(2) Ideal state:

Prepare raw materials according to the aforementioned test group. First, extract and screen RAP into the binder, filler, and aggregate. Place these components together with virgin asphalt and virgin aggregate into a constant-temperature oven for preheating to 150 °C. Place both virgin and recycled aggregates into a 150 °C mixing drum for 90 s of mixing. Subsequently, add the binder and virgin asphalt for 90 s of mixing. Finally, incorporate the filler and complete 90 s of mixing. Maintain a mixing temperature of 150 °C throughout the entire process, with a total mixing duration of 270 s.

#### 2.2.2. Separating Virgin and Aged Materials

To separate aged and virgin aggregate from the recycled mixture, the construction process based on in situ thermal recycling replaces virgin aggregate of 4.75 mm and larger size with magnetite aggregate. Virgin asphalt, virgin aggregates, and RAP are mixed to form a recycled asphalt mixture, dispersed into a colloidal state, and then separated using magnetic attraction to isolate magnetite aggregates from the RAP. The separated magnetite aggregates retain residual fine aged particles and asphalt film.

**Figure 6:**
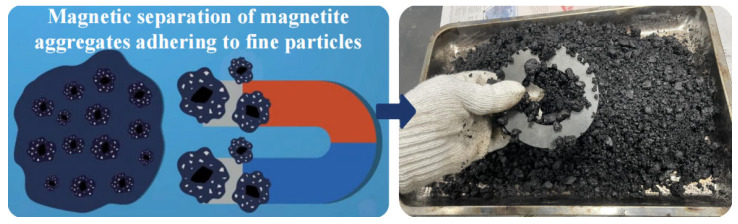
Separation of virgin and aged coarse aggregates.

The prepared thermally recycled asphalt mixture was evenly spread and dispersed on a dedicated test bench. An infrared thermometer monitored the mixture’s temperature until it reached approximately 70 ± 2 °C. At this point, a magnet was swiftly applied to separate the recycled material from the magnetite aggregate, as shown in Figure 6.

#### 2.2.3. Separating Asphalt from Aggregates

**Figure 7:**
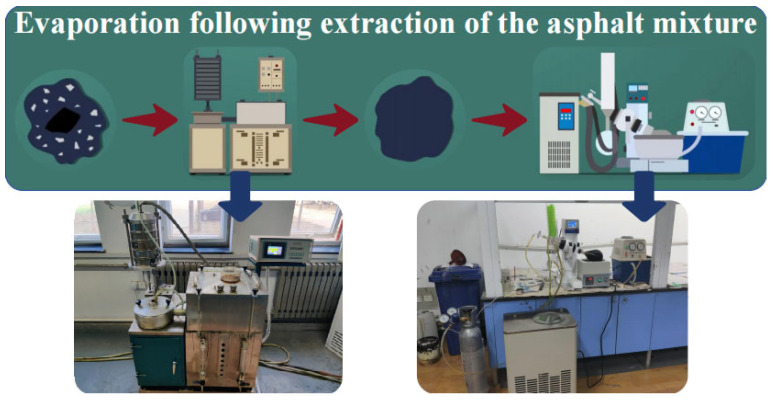
Separation of asphalt and aggregates.

Using an extractor, the separated magnetite aggregate was processed. After separating the residual fine aged material particles from the asphalt binder, the separated fine aged material particles were screened to determine their mass. Parallel tests were conducted according to this procedure, and the results were based on the arithmetic mean of the two sets of data, as shown in Figure 7.

#### 2.2.4. Calculating the Blending Degree

The degree of mixing between virgin and aged aggregates is quantified as the uniformity $\lambda_{i}$ of particles adhering to the surface of magnetite aggregates. During actual mixing, the mass $m_{i}$ of fine-grained mineral particles adhering to the surface of magnetite aggregates represents each specification. Taking the ideal state—where thorough mixing occurs—as the benchmark, the mass $m_{i}^{*}$ of fine-grained mineral particles adhering to the surface of magnetite aggregates serves as the reference value. This enables the determination of mixing uniformity under varying degrees of mixing, then $\lambda_{i}$ is calculated as per Formula (1).

(1) $$\lambda_{i}=\frac{m_{i}}{m_{i}^{*}}\times 100\%$$

### 2.3. Experimental Design

To analyse the effects of various factors on the mixing state of new and old aggregate particles, in accordance with the Chinese specifications under the Technical Specification for Construction of Highway Asphalt Pavements (JTG F40-2004) [31], this paper employs the Marshall mix design method to calculate the optimal asphalt content in accordance with the AC-20 plant-mixed hot-recycled standard. The gradation range of the mixture is shown in Table 5.

Multiple factors influence the degree of blending between virgin and aged aggregates during hot central plant recycling. Drawing on extensive engineering practice experience [32], this study investigates six key influencing factors: amount of RAP, amount of rejuvenating agent, mixing temperature, mixing time, mixing orders, and asphalt content. The amount of RAP refers to the percentage of RAP in the total mass of the hot-recycled asphalt mixture. This experiment employed RAP content levels of 20%, 30%, 40%, and 50%, with the mix proportions for each test group as shown in Table 6.

The amount of rejuvenating agent is calculated based on the mass of aged asphalt. Mixing temperature encompasses the asphalt heating temperature, the temperature maintained during mixing, and the discharge temperature to be controlled. Mixing time refers to the total mixing duration of virgin and aged aggregates with the binder. Mixing orders involve blending RAP, virgin asphalt, and virgin aggregates in different orders.

**Figure 8:**
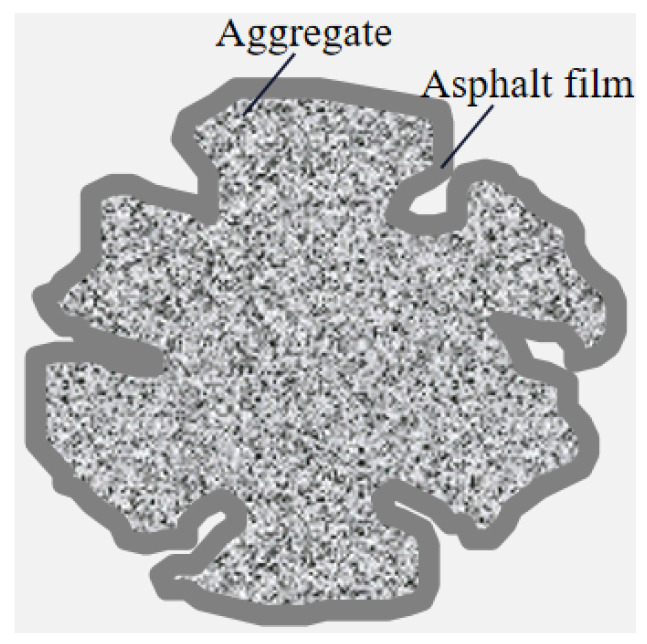
Asphalt film schematic diagram.

As illustrated in Figure 8, the total asphalt content includes both virgin and aged asphalt. For convenience in calculation, this paper uses the oil film thickness of the aggregate as the control parameter. Given the oil film thickness, the required virgin asphalt content in the mixture is calculated as follows:

(1) First, determine and calculate the synthetic bulk density of the ore material, then compute its specific surface area *SA* [33], according to Equation (2).

(2) $$SA={FA}_{i}\times P_{i}$$

where *SA* is the surface area of the aggregate, m^2^/kg; ${FA}_{i}$ is the surface area factor for aggregates of each particle size; and *P_i_* indicates the percentage passing rate for each particle size, %.

(2) Determine the maximum theoretical density of the mixture using the vacuum method. Calculate the synthetic effective density and effective asphalt content of the mineral aggregate. Finally, calculate the required asphalt mass *m_b_* for mixing the mixture according to Equation (3).

(3) $$m_{b}=\frac{DA\times SA\times \gamma_{b}}{\omega_{be}}\times m_{s}$$

where *SA* denotes the specific surface area of the aggregate, m^2^/kg; *DA* represents the effective thickness of the asphalt film, μm; $\gamma_{b}$ is the relative density of asphalt (25 °C/25 °C); $\omega_{be}$ is the mass fraction of effective asphalt, %; *m_s_* is the mass of mineral aggregate, kg.

(3) Based on Equations (2) and (3), calculate the total asphalt mass required by determining the oil film thickness for all aggregate materials (both virgin and aged aggregates). Subtract the asphalt mass already present in the RAP to obtain the amount of virgin asphalt that needs to be added during the mixing process.

The influencing factors and level settings selected for this study are shown in Table 7.

## 3. Results and Discussion

### 3.1. Effect of Experimental Parameters on Degree of Blending

#### 3.1.1. Effect of Amount of RAP and Rejuvenating Agent

**Figure 9:**
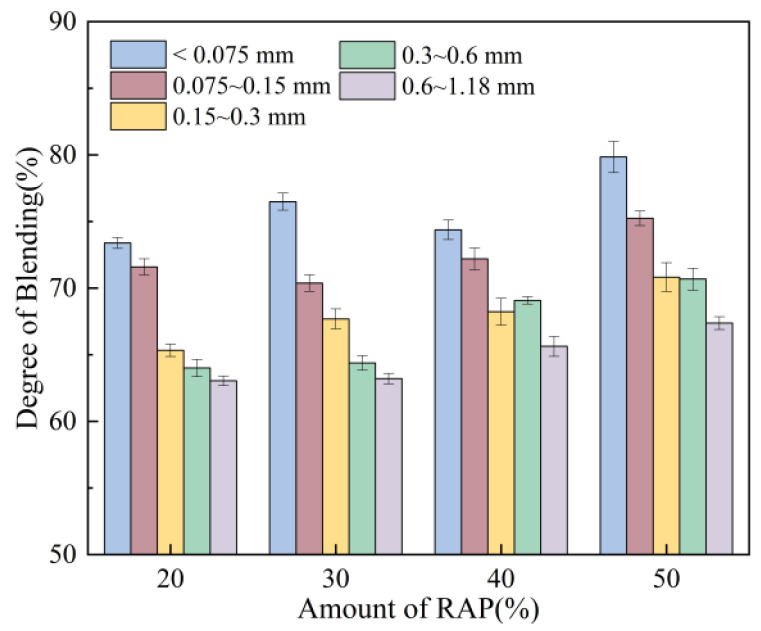
Effect of amount of RAP on degree of blending.

The effects of the amount of RAP are shown in Figure 9. Under identical conditions, the coarse particles in thermally recycled asphalt mixtures exhibit significantly less fine aggregate coating compared to conventional asphalt mixtures. Fine particles (≤1.18 mm) in RAP failed to fully separate from the larger-sized recycled aggregate, achieving a blending degree of approximately 60% to 80%. This indicates that under identical recycling conditions, the amount of RAP does not significantly affect the blending degree between virgin and aged aggregate particles. At low RAP content, the mixing process primarily involves adhesion between virgin aggregate and virgin asphalt. Aged aggregate in the RAP remains largely bound to aged asphalt and fails to participate in mixing, resulting in minimal aged aggregate on magnetite aggregate and consequently low blending degree. As the amount of RAP increases, more aged aggregate can participate in the mixing process, enhancing the degree of blending. However, excessively high content may prevent thorough mixing or cause a decrease in mixing temperature, slowing down the mixing process.

**Figure 10:**
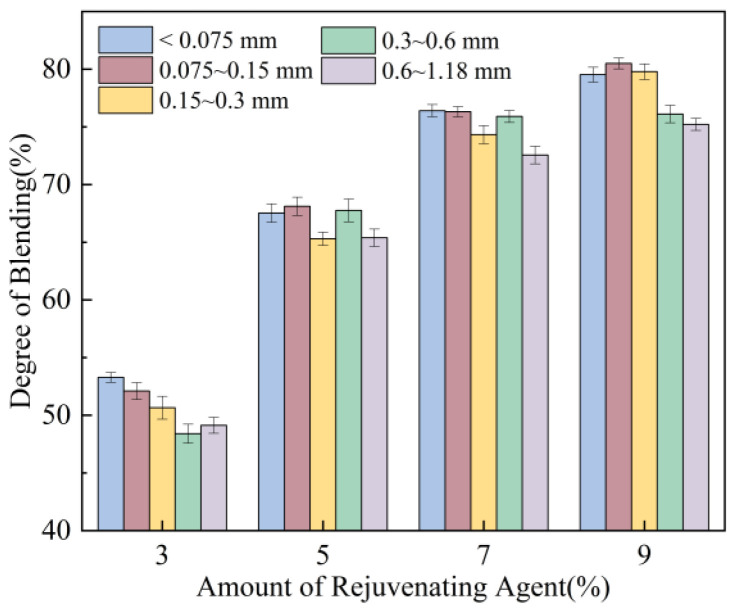
Effect of amount of rejuvenating agent on degree of blending.

As shown in Figure 10, the degree of blending between virgin and aged aggregates progressively improves with increasing amount of rejuvenating agent. However, beyond 7%, the blending efficiency shows little further change, stabilising at approximately 70% to 80%. As rejuvenating agents soften aged asphalt to enhance its fluidity, they significantly improve the separation of asphalt and aggregate within RAP. The addition of a rejuvenating agent improves the blending efficiency of virgin and aged mineral aggregates of varying specifications by 30% to 40%. This occurs because the rejuvenating agent reacts with the aged asphalt in RAP, whose viscosity has markedly improved. This reaction effectively reduces the viscosity of the aged asphalt and enhances its fluidity, thereby facilitating the blending of virgin and aged mineral particles. This demonstrates that appropriately improving the amount of rejuvenating agent can improve the fusion between virgin and aged asphalt in thermally recycled asphalt mixtures, thus ensuring the uniformity of mixing in thermally recycled asphalt mixtures.

#### 3.1.2. Effect of Mixing Temperature and Mixing Time

**Figure 11:**
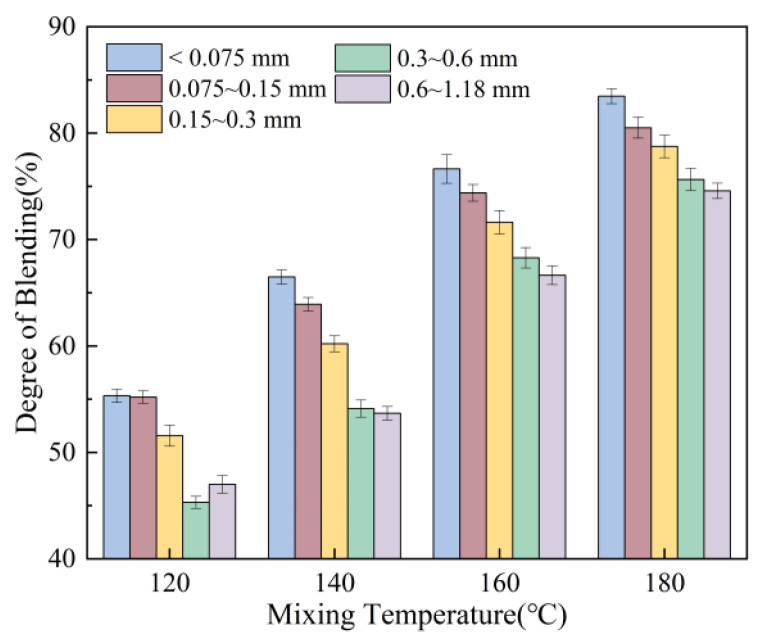
Effect of mixing temperature on degree of blending.

Figure 11 illustrates the effect of mixing temperature. As the mixing temperature increases during the recycling process, the degree of blending of the aged aggregate improves. At a mixing temperature of 180 °C, the blending degree reaches approximately 80%. Test results indicate that the degree of blending between virgin and aged mineral particles exhibits a gradual increase trend with rising mixing temperatures. Therefore, elevating the mixing temperature of thermally recycled asphalt mixtures can enhance the blending degree by 10% to 30%, effectively promoting the separation of fine particles from the aged mineral aggregate within the coarse mineral particles of RAP. This occurs because elevated mixing temperatures enhance binder fluidity and reduce viscosity. This facilitates mixing between fine particles of recycled aggregate and virgin aggregate particles, breaks up potential aggregate clumps, and minimises segregation between different particle sizes. Consequently, it ensures uniform distribution of all components within the mixture, aligning the actual gradation more closely with the design gradation.

**Figure 12:**
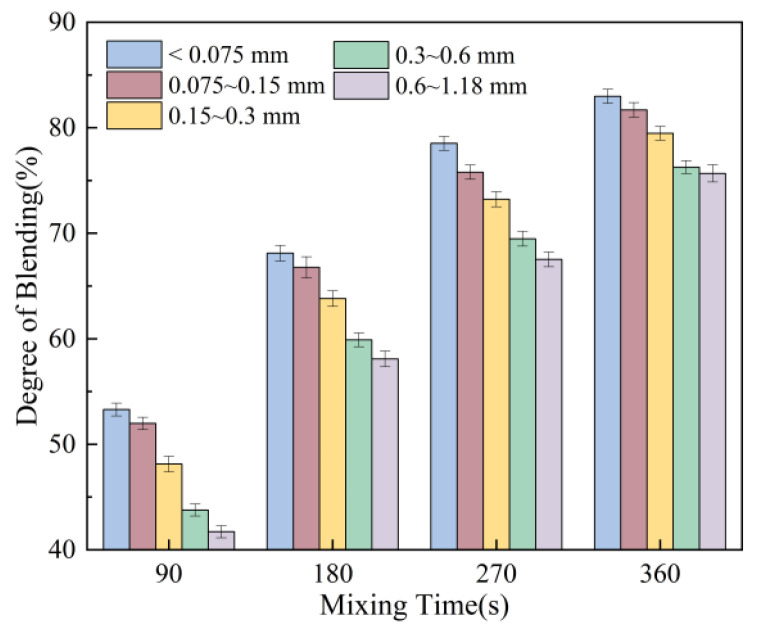
Effect of mixing time on degree of blending.

The effect of mixing time is shown in Figure 12. When the mixing time is 90 s, the blending degree of the recycled aggregate is approximately 50%. As the mixing time increases from 90 s to 360 s, the blending degree rises from 50% to 80%. This phenomenon primarily occurs because extended mixing time provides the mixing equipment with more operational time to break apart lumps or agglomerates within the RAP, thereby releasing more fine particles from the aged aggregate and promoting their uniform distribution within the mixture. However, once the mixing time reaches a certain threshold, the mixture gradually achieves a state of dynamic equilibrium. At this point, the mixing and redistribution of virgin and aged aggregate particles stabilise, limiting the effectiveness of further extending mixing time to improve blending. However, when the mixing temperature reaches 180 °C and the mixing time is 360 s, blending exceeds 80%. This is attributed to excessive mixing temperature and duration, causing the secondary ageing of the asphalt and distorting the calculated results.

#### 3.1.3. Effect of Mixing Order and Asphalt Content

**Figure 13:**
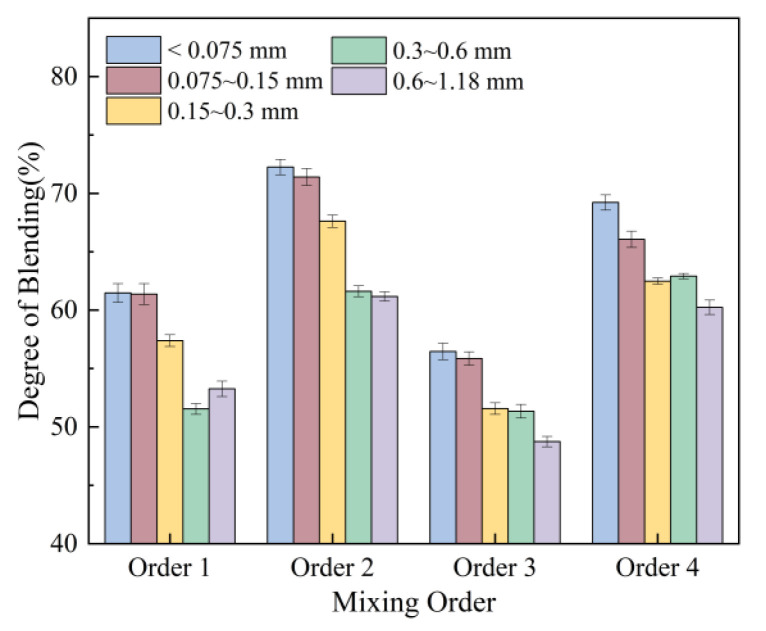
Effect of mixing order on degree of blending.

The results of mixing sequence effects are shown in Figure 13. The mixing sequence during the thermal recycling process significantly impacts the degree of blending. When RAP is mixed with virgin aggregate first, the mineral aggregate blending degree can reach 60% to 70%. This occurs because mixing RAP with virgin aggregate first allows earlier and more extensive contact between virgin and aged aggregates, thereby enhancing the blending degree.

**Figure 14:**
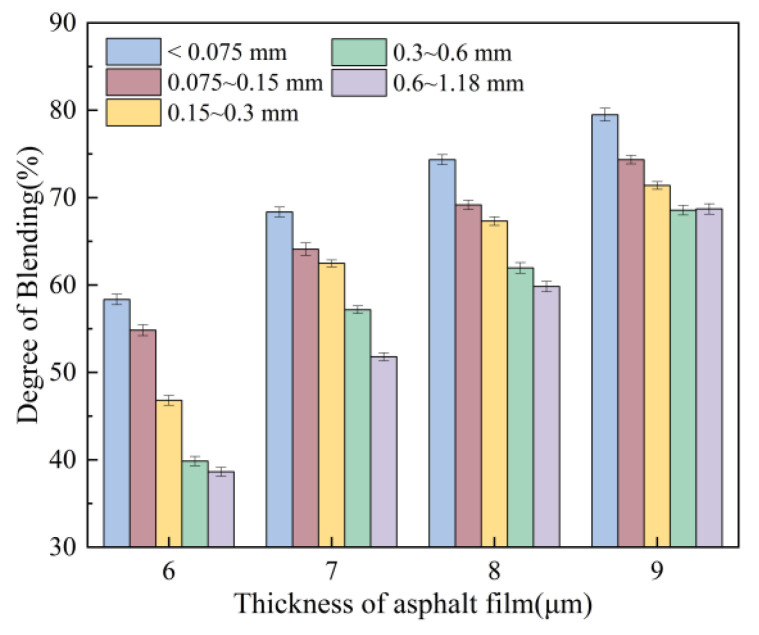
Effect of asphalt film thickness on degree of blending.

Figure 14 shows that as the asphalt content increases, the degree of blending between different-sized recycled aggregate particles and fresh aggregate gradually rises from 40% to approximately 70%. Analysis indicates that this occurs because the aged asphalt in RAP becomes viscous, while the added fresh asphalt reduces this viscosity by blending with it, enhancing flowability and thereby promoting the mixing of virgin and aged aggregate particles. This effect becomes more pronounced with increased fresh asphalt content. However, during testing, asphalt mixtures prepared with a 9 μm oil film thickness exhibited minor oil bleeding, precluding further increases in asphalt content.

### 3.2. Analysis of Variance

Based on the aforementioned experimental data, statistical analysis was conducted using IBM SPSS Statistics 27 software to determine the significance of each influencing factor on the degree of blending between the virgin and aged aggregate. The results of the analysis of variance are presented in Table 8.

As shown in Table 8, the significance probability of the F-value corresponding to the amount of RAP is 0.441, which is greater than 0.05. This indicates that this factor does not significantly affect the blending degree. It provides a theoretical basis for increasing the amount of RAP within permissible limits, thereby reducing the cost of virgin materials without compromising mixing uniformity. The significance levels of all other experimental parameters are less than 0.05, indicating a significant effect on the degree of blending between the virgin and aged aggregates. Mixing temperature directly affects the viscosity and fluidity of asphalt. Identifying a temperature sufficient to ensure adequate mixing prevents energy wastage or asphalt ageing, thereby safeguarding long-term performance. The function of the rejuvenating agent is to permeate and soften aged RAP asphalt, while the mixing sequence determines whether the rejuvenating agent and new/old materials achieve sufficient contact, and mixing time directly relates to production efficiency and energy consumption. Determining the optimal time through experimentation maximises output per unit time, thereby directly enhancing production capacity and profitability. Asphalt content significantly influences nearly all properties of the mixture, including mix homogeneity, durability, rutting resistance, and resistance to water damage. The factors are ranked in the order of their impact based on the experimental results as follows: mixing time > amount of rejuvenating agent > mixing temperature > asphalt content > mixing order > amount of RAP. It is worth noting that mixing time has the most significant impact on the blending degree of virgin and aged aggregate materials.

**Figure 15:**
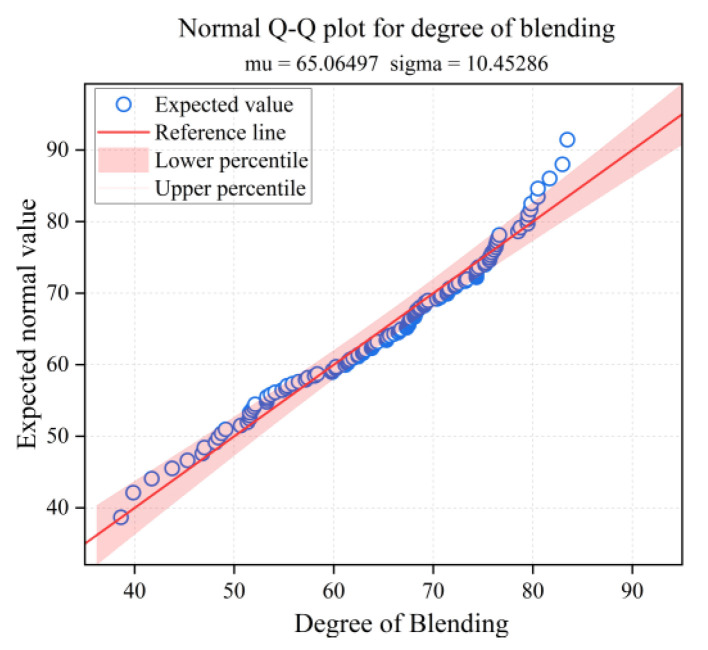
Normal Q–Q plot for degree of blending.

**Figure 16:**
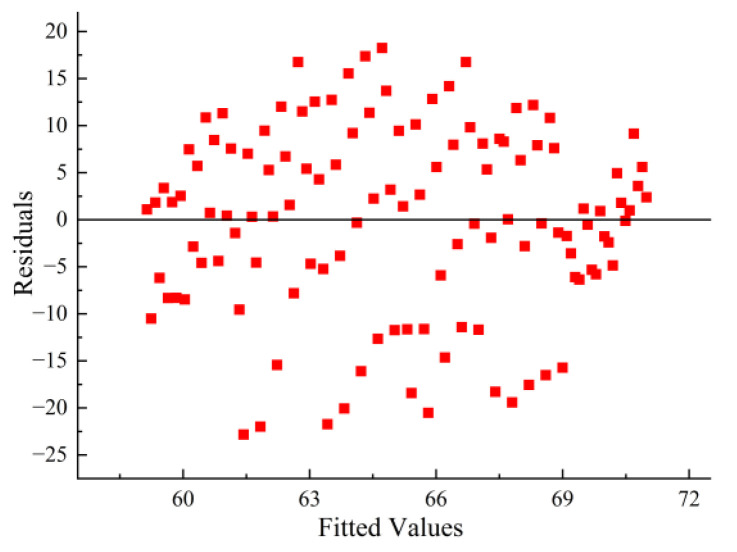
Residuals vs. fitted values.

To ensure the reliability of the analysis of variance results, residual diagnostics were conducted. As shown in Figure 15, the standard Q–Q plot for the degree of blending reveals points distributed along the diagonal, indicating that the normality assumption is satisfied mainly. Figure 16 shows the residuals versus fitted values plot, where the points are randomly scattered around the zero line with no discernible pattern, supporting the assumption of homoscedasticity. As experiments were conducted in random order, the independence assumption holds. In summary, the model assumptions are reasonably satisfied, rendering the aforementioned statistical inference reliable.

### 3.3. Blending Rules of Virgin and Aged Aggregates

During hot central plant recycling, the degree of blending between virgin and aged aggregates is influenced by multiple factors, with each factor exhibiting a specific pattern of effect on the mixing process. As the amount of RAP, the amount of rejuvenating agent, mixing temperature, mixing time, mixing orders, and asphalt content increase, the degree of blending between virgin and aged aggregates shows an upward trend. Optimal blending is achieved when materials are added in the sequence: RAP, virgin aggregate, and virgin asphalt. The mixing rules for virgin and aged aggregates are detailed in Table 9.

Based on test results, the variation in the aforementioned influencing factors must be controlled to enhance the blending of virgin and aged aggregates. During the production of recycled asphalt mixtures, increasing the mixing temperature correspondingly extends the mixing time, which improves the blending of virgin and aged aggregates. However, excessive increases in mixing temperature and time may lead to asphalt ageing [34]. Therefore, all influencing factors should be adjusted within reasonable limits to enhance the uniformity of blending between virgin and aged aggregates.

## 4. Conclusions

This study employed magnetite to replace virgin aggregates in the 5 mm to 20 mm particle size range. A novel methodology was developed encompassing asphalt mixture preparation, separation of virgin and aged materials, separation of asphalt and mineral aggregates, and calculation of blending degree. Furthermore, it analyses the influence patterns of various factors on the blending degree between virgin and aged aggregates. Key conclusions are as follows:

(1) Under normal mixing conditions, virgin and aged aggregates cannot achieve 100% blending, with a blending degree of only 40% to 60%, failing to meet design expectations. The test results confirm that adjusting the aforementioned influencing factors can increase the blending degree of virgin and aged aggregates to 60% to 80%.

(2) Increasing the amount of RAP during plant-mixed hot recycling has a negligible effect on blending efficiency. However, appropriately increasing the amount of rejuvenating agent, mixing temperature, mixing time, and asphalt content can correspondingly improve blending efficiency by 10% to 30%. Additionally, the mixing sequence, where RAP is first blended with virgin aggregate before adding virgin asphalt, can enhance the blending efficiency of virgin and aged aggregates by approximately 20%. The relative importance of factors is as follows: mixing time > amount of rejuvenating agent > mixing temperature > asphalt content > mixing orders > RAP dosage.

(3) A novel experimental method has been proposed to quantify the degree of blending between virgin and aged aggregates. Similar to the research methodology employed by Ma Tao et al. mentioned earlier in this paper, comparative tests were conducted using other experiments as control groups, where thorough mixing was considered the ideal state. This experimental methodology encompasses key steps, including the asphalt mixture preparation, separation of virgin and aged materials, separation of binder and mineral aggregate, and calculation of blending degree. Experimental validation confirms the method’s capability to accurately and consistently quantify the blending degree of virgin and aged aggregates, thereby elucidating the blending behaviour of virgin and aged aggregates within thermally recycled asphalt mixtures.

This study analysed the dispersion uniformity of fine mineral aggregates in RAP during mixing by comparing their movement characteristics under actual and fully mixed conditions. Subsequent research will adjust asphalt content, aggregate gradation, and mixing parameters based on measured blending levels, thereby applying the blending degree of virgin and aged aggregates as a parameter to enhance the service life and performance of recycled mixtures.

## Figures and Tables

**Table 1:** Performance indicators of virgin asphalt.

Testing Index	Test Method [29]	Technical Requirements	Virgin Asphalt
Penetration (25 °C, 100 g, 5 s, 10^−1^ mm)	T 0604-2011	60~80	71.3
Ductility (5 cm/min, 10 °C, cm)	T 0605-2011	≥25	34.5
Softening point (ring and ball method, °C)	T 0606-2011	≥46	50.7
Density (15 °C, g·cm^−3^)	T 0603-2011	—	1.048

**Table 2:** Performance index of magnetite coarse aggregates.

Testing Index	Technical Requirements	Test Results
Crushing value (%)	≤28	10.2
Los Angeles coefficient (%)	≤30	8.9
Apparent specific gravity (g/cm^3^)	≥2.5	3.91
Water absorption (%)	≤3.0	0.34
Ruggedness (%)	≤12	0.3
Adhesion to asphalt	≥4	Level 5
Polished stone value	≥42	44

**Table 3:** Performance indicators of aged asphalt.

Testing Index	Test Method [29]	Technical Requirements	Aged Asphalt
Penetration (25 °C, 100 g, 5 s, 10^−1^ mm)	T 0604-2011	60~80	30.8
Ductility (5 cm/min, 10 °C, cm)	T 0605-2011	≥25	2.3
Softening point (ring and ball method, °C)	T 0606-2011	≥46	66.4
Density (15 °C, g·cm^−3^)	T 0603-2011	—	1.042

**Table 4:** Material gradation for each grade.

Screen Hole Diameter	10~20 mm	5~10 mm	0~5 mm	Fillers	0~10 mm RAP	10~20 mm RAP
26.5	100	100	100	100	100	100
19	90.3	100	100	100	100	100
16	62.7	100	100	100	100	97.4
13.2	29.5	100	100	100	100	91.6
9.5	3.2	90.1	100	100	90.8	60.0
4.75	0.4	13.5	98.7	100	53.2	34.4
2.36	0.4	2.6	72.3	100	26.4	18.2
1.18	0.3	0.6	53.0	100	17.4	13.9
0.6	0.3	0.6	32.6	100	11.6	9.2
0.3	0.3	0.6	18.7	100	8.0	7.6
0.15	0.3	0.5	11.8	100	6.4	6.2
0.075	0.2	0.4	3.8	90.6	4.7	5.0

**Table 5:** Gradation range.

**Sieve Mesh** **(mm)**	26.5	19.0	16.0	13.2	9.5	4.75	2.36	1.18	0.6	0.3	0.15	0.075
**Aggregate passing rate**	100	90	78	62	50	26	16	12	8	5	4	3
		~	~	~	~	~	~	~	~	~	~	~
		100	92	80	72	52	44	33	24	17	13	7

**Table 6:** Aggregate gradation for different amounts of RAP.

Aggregate	10–20 mm	5–10 mm	0–5 mm	Filler	0–10 mm RAP	10–20 mm RAP
20% RAP	35	14	27	4	10	10
30% RAP	32	11	23	4	15	15
40% RAP	28	8	20	4	20	20
50% RAP	28	5	13	4	25	25

**Table 7:** Level setting of experimental parameters.

Test Group	Amount of RAP (%)	Amount of Rejuvenating Agent (%)	Mixing Temperature (°C)	Mixing Time (s)	Mixing Orders	Thickness of Asphalt Film (μm)
1	20	3	120	90	Order 1	6
2	30	5	140	180	Order 2	7
3	40	7	160	270	Order 3	8
4	50	9	180	360	Order 4	9

Note: Order 1 involves mixing RAP with virgin asphalt first, followed by incorporation of virgin aggregate. Order 2 involves mixing RAP with virgin aggregate first, followed by incorporation of virgin asphalt. Order 3 involves mixing virgin asphalt with virgin aggregate first, followed by incorporation of RAP. Order 4 involves simultaneous mixing of all three components.

**Table 8:** Analysis of variance for the effect of each parameter on the degree of blending.

Dependent Variable: Degree of Blending						
Source	Type III SS	DF	MS	F	P	ηp^2^
Calibration model	2388.369 ^a^	22	108.562	5.721	0.019	0.954
Intercept	308.414	1	308.414	16.253	0.007	0.730
Particle size	59.103	3	19.701	1.038	0.045	0.342
Amount of RAP	501.688	3	167.229	8.813	0.441	0.815
Amount of rejuvenating agent	475.414	3	158.471	8.351	0.013	0.807
Mixing temperature	535.624	3	178.541	9.409	0.015	0.825
Mixing time	330.04	3	110.013	5.798	0.011	0.744
Mixing order	457.861	3	152.62	8.043	0.033	0.801
Asphalt content	361.744	4	90.436	4.766	0.016	0.761
Error	113.854	6	18.976	—	—	—
Grand total	120,179.055	29	—	—	—	—
Total correction	2502.223	28	—	—	—	—

R Squared = 0.954 (Adjusted R Squared = 0.788). ^a^ This implies that all independent variables (factors) included in the model can significantly account for the variation in the dependent variable.

**Table 9:** Influence of different factors on the degree of blending.

Influencing Factor	Trends in Influencing Factors	Trends in the Degree of Blending	Ranking of Influencing Factors
Amount of RAP	Increase	Enhance	6
Amount of rejuvenating agent	Increase	Enhance	2
Mixing temperature	Increase	Enhance	3
Mixing time	Increase	Enhance	1
Mixing orders	RAP + virgin aggregate + Virgin asphalt	Enhance	5
Asphalt content	Increase	Enhance	4

## Data Availability

The original contributions presented in this study are included in the article. Further inquiries can be directed to the corresponding authors.
